# Information Integration and Information Storage in Retinotopic and Non-Retinotopic Sensory Memory

**DOI:** 10.3390/vision5040061

**Published:** 2021-12-13

**Authors:** Haluk Öğmen, Michael H. Herzog

**Affiliations:** Department of Electrical & Computer Engineering, University of Denver, Denver, CO 80208, USA; Laboratory of Psychophysics, Brain Mind Institute, Ecole Polytechnique Fédérale de Lausanne (EPFL), 1015 Lausanne, Switzerland; michael.herzog@epfl.ch

**Keywords:** temporal dynamics, iconic memory, retinotopy, retinotopic reference-frame, non-retinotopic reference-frame, ecological vision

## Abstract

The first stage of the Atkinson–Shiffrin model of human memory is a sensory memory (SM). The visual component of the SM was shown to operate within a retinotopic reference frame. However, a retinotopic SM (*r*SM) is unable to account for vision under natural viewing conditions because, for example, motion information needs to be analyzed across space and time. For this reason, the SM store of the Atkinson–Shiffrin model has been extended to include a non-retinotopic component (*nr*SM). In this paper, we analyze findings from two experimental paradigms and show drastically different properties of *r*SM and *nr*SM. We show that *nr*SM involves complex processes such as motion-based reference frames and Gestalt grouping, which establish object identities across space and time. We also describe a quantitative model for *nr*SM and show drastic differences between the spatio-temporal properties of *r*SM and *nr*SM. Since the reference-frame of the latter is non-retinotopic and motion-stream based, we suggest that the spatiotemporal properties of the *nr*SM are in accordance with the spatiotemporal properties of the motion system. Overall, these findings indicate that, unlike the traditional *r*SM, which is a relatively passive store, *nr*SM exhibits sophisticated processing properties to manage the complexities of ecological perception.

## 1. Introduction

**Figure 1:**
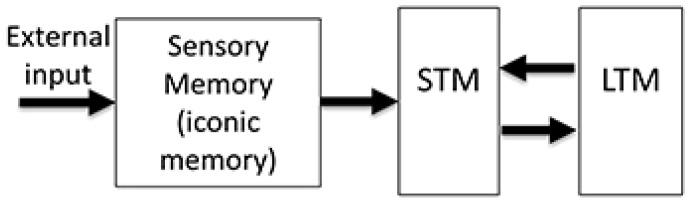
Atkinson Shiffrin model. It is a multi-store model consisting of Sensory Memory (SM), Short-Term Memory (STM), and Long-Term Memory (LTM). Each store has distinct properties. From Ref. [3].

Human memory can be described by three distinct stores [1] (Figure 1). The first stage is called Sensory Memory (SM), which has a very large capacity but its contents decay relatively fast (within few hundred milliseconds). The second stage is Short-Term Memory (STM) or Working Memory (WM). Information in the STM/WM can be held for longer periods of time, in the order of several seconds. Still, the capacity of STM/WM is severely limited and only few items can be stored simultaneously. Finally, the last stage is Long-Term Memory (LTM), which has a very large capacity and can hold information as long as a lifetime. Since all information stored in STM/WM as well as LTM originates from SM, understanding how SM operates is essential for understanding human memory and learning processes. Although there a large number of studies have been conducted, the way in which SM operates under ecological viewing-conditions remains an unsolved puzzle [2]. For example, in laboratory experiments with static stimuli, it was shown that the reference frame for SM is retinotopic, i.e., anchored on the eyes. Note that when the stimulus is static with respect to the observer, retinotopic and non-retinotopic reference-frames cannot be distinguished from each other; hence, experiments with static stimuli do not necessarily reveal exclusive properties of retinotopic reference-frames. However, as we will show in the rest of the manuscript, when the stimulus is in motion, one can distinguish between retinotopic and non-retinotopic reference-frames. Under ecological viewing conditions, our eyes, head, body, and many external objects are in motion. A retinotopically based memory system would create highly smeared and improperly superimposed contents, which do not correspond to our phenomenal experience. Indeed, under ecological viewing, objects appear according to their spatial and not retinotopic coordinates and our perception is generally sharp and clear.

**Figure 2:**
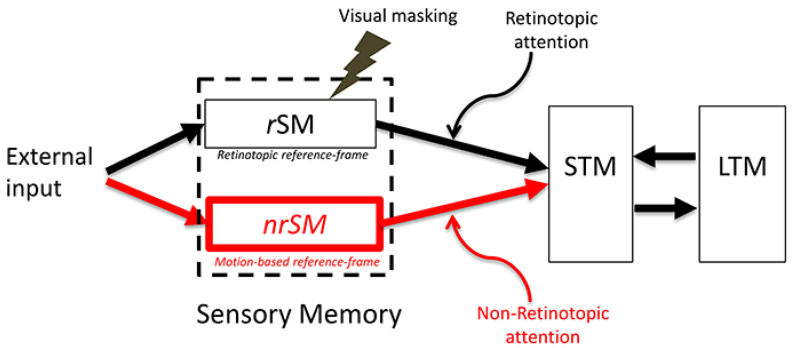
The modified SM model. In addition to the traditional retinotopic SM (*r*SM) in the Atkinson–Shiffrin model, a second non-retinotopic component, *nr*SM is added. From Ref. [3].

In the last two decades, a modified model of SM was proposed [3], as shown in Figure 2.

This modified model divides SM into two components: *r*SM and *nr*SM, a retinotopic and a non-retinotopic sensory memory, respectively. A major difference between these two components is their reference frame, whereby *r*SM and *nr*SM use retinotopic and non-retinotopic reference frames, respectively. The former corresponds to the SM characterized by static stimuli used in earlier studies whereas the latter component explains how moving object-information is stored in memory.

In this paper, we analyze findings from two experimental paradigms, the Sequential Metacontrast and Ternus-Pikler display (TPD), to show drastically different properties of *r*SM and *nr*SM. Although it is well known that retinotopically overlapping stimuli are integrated in the *r*SM, the integration is non-retinotopic in *nr*SM. Furthermore, this non-retinotopic integration is specific to motion streams and to object identities established by Gestalt grouping. This is a drastic departure from *r*SM, which is taught to be independent of operations such as Gestalt grouping. The operation of Gestalt grouping at such an early stage challenges the view of grouping as a high-level process. Furthermore, we provide evidence supporting the view that non-retinotopic integration does not stem from high-level decision process weighing and by integrating different response options. Rather, non-retinotopic integration is an unconscious process combining information linearly with accuracy. The contents of *r*SM are susceptible to visual masking while those of *nr*SM are not [4]. Here, we illustrate an implication of this property by examining the operation of *nr*SM across saccades. We also provide a quantitative model describing the operation of *nr*SM and showing drastic differences between spatio-temporal properties of *r*SM and *nr*SM. Since the reference-frame of the latter is non-retinotopic and motion-stream based, we suggest that spatiotemporal properties of *nr*SM are in accordance with the spatiotemporal properties of the motion system. Overall, these findings indicate that, unlike the traditional rSM, which is a relatively passive store, *nr*SM exhibits sophisticated processing properties to manage the complexities of ecological perception.

## 2. Sequential Metacontrast and Feature Processing in Space and Time

### 2.1. Sequential Metacontrast

Sequential metacontrast is a specific type of visual masking. Visual masking refers to the reduced visibility of a target due to the presence of a spatiotemporally proximal mask [5,6]. This phenomenon has been used extensively to study the spatiotemporal dynamics of visual processing and memory. Typically, the target and the mask stimuli are presented briefly in time and the target’s visibility is measured as a function of the temporal asynchrony between their onsets (SOA: stimulus onset asynchrony). Metacontrast masking is of special interest, where the target and the trailing mask do not overlap spatially, because of the retroactive effect the mask exerts on the target. Sequential metacontrast [7,8] consists of repetitive application during the time of masking.

### 2.2. Visible Features of Invisible Targets

**Figure 3:**
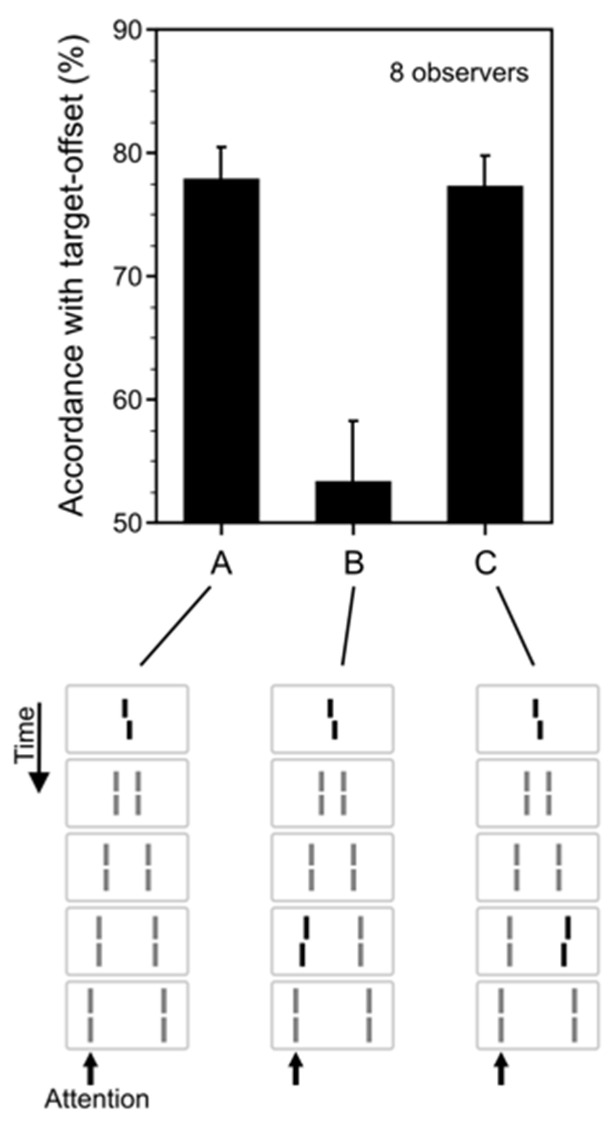
Sequential Metacontrast. A central line is followed by flanking lines at distances increasingly further away in space. In the experiments, observers attended, say, to the left stream and reported the perceived offset of the elements in the left stream. The reported offset direction was largely in agreement with the offset direction of the invisible central line (**A**). Hence, an invisible element can transfer its features to visible elements in a rather predictable manner. When one of the lines was offset in the opposite direction than the central vernier, the two offsets cancelled (**B**). When the line was offset in the non-attended, right stream, no integration occurred (**C**). Integration occurs only within a stream, i.e., a perceptual group. From Ref. [8].

Figure 3 shows the sequential metacontrast stimulus used by Otto et al. [8]. A central line with a Vernier offset is flanked by pairs of lines presented increasingly distant positions. Observers perceive a stream of lines originating from the center. The central line is rendered invisible by metacontrast masking, i.e., it is largely unconscious. To measure the visibility of the central line, Otto et al. ran a 2IFC experiment, where the central line was either absent or present and observers indicated which interval the central line was present in. Performance was close to chance level [8]. Interestingly, in the next experiment, when the central line had a Vernier offset, all subsequent lines appeared as offset in the same direction as the central line offset—even though the central line was invisible.

### 2.3. Non-Retinotopic Feature Integration and Stream Specificity

As shown in Figure 3B, when a Vernier offset with an opposite direction (called “anti-Vernier” hereafter) to the offset of the central Vernier is inserted to the stream, the agreement of observers’ responses with the direction of the central Vernier becomes close to 50%, indicating a summation of the two Verniers in the stream. Furthermore, an inspection of Figure 3C reveals that this summation is stream-specific. When the anti-Vernier is inserted to the right stream, instead of the left stream for which observers report the perceived Vernier offset, it no longer sums and cancels the effect of the central Vernier, as indicated by the similar levels of accordance in Figure 3A,C. Conversely, it is known that two stimuli presented at the same retinotopic location in close temporal succession integrate [9,10,11,12,13,14], and these results show an integration that is non-retinotopic since the Vernier and the anti-Vernier are presented at different retinotopic locations. However, not any two arbitrary stimuli can be integrated; for integration to take place, the stimuli have to belong to the same motion stream, i.e., to the same object.

In summary, unlike the “passive” retinotopic integration in *r*SM, integration in *nr*SM is more complex and includes motion-related processes to determine which stimuli across space and time get integrated.

### 2.4. Feature Integration Is Approximately Linear

**Figure 4:**
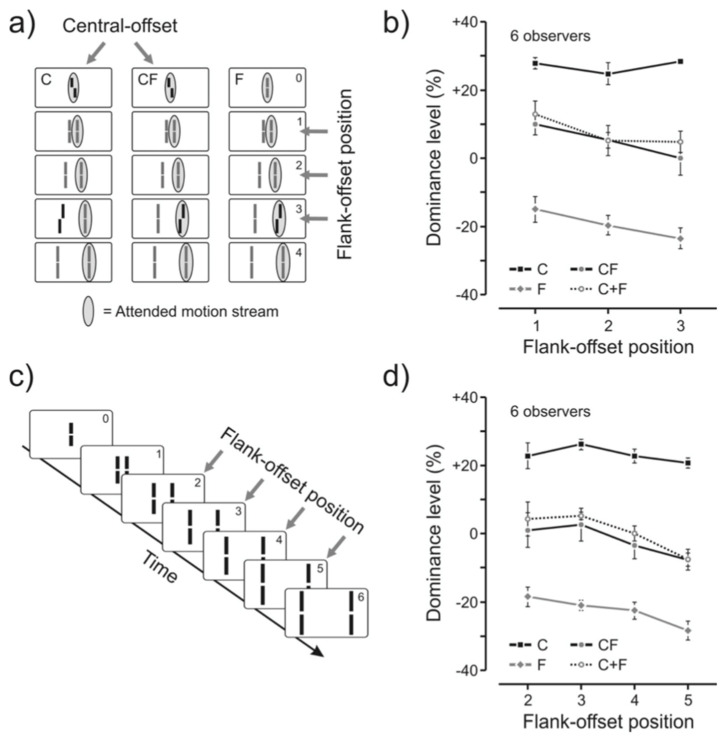
(**a**) Same paradigm as in the experiment before, with the exception that observers attended to the right stream. (**b**) A line (Flank) was offset in the opposite direction to the central vernier, which could be in the non-attended, left stream (condition C), in the attended, right stream (condition CF). In condition F, the central line was not offset. We varied the line/flank position for all conditions (**c**). (**b**) Dominance indicates to what extent the central offset determines the response. Values below indicate that line/flank offset dominates the response. If the flank offset is in the non-attended stream, dominance stays roughly constant because, as in the previous figure, it does not contribute to performance (condition C). If the central line is not offset, the line/flank offset determines performance the stronger the latter it comes (condition F). The effect is strongest for the last position. Combining conditions C and F (condition CF), leads to a dominance level, which is well described by a simple average of the two conditions (C + F). Hence, performance is roughly linear. (**d**) Same experiment as in (**b**) for the stimulus shown in panel (**c**) with flank-offset positions varying between 2 and 5. From Ref. [14].

In order to investigate the nature of feature integration further, Otto et al. [14] used the concept of “dominance level” to quantify the contributions of various Vernier offsets to the final perception (Figure 4). The left panel of Figure 4 shows a central Vernier (C), and a flank anti-Vernier (F). In this case, F does not belong to the attended stream (indicated by gray ellipses) and hence does not integrate with C. The percentage of accordance with the offset direction of C indicates the contribution of C alone to the final percept. In the right most panel, C does not contain any offset and F is added to the attended stream. This condition attempts to measure the contribution of F alone to the final percept. Finally, in the middle panel, C and F are both inserted to the attended stream to measure their combined (integrated) effect. The accordance scale ranges from 0% to 100%, and an accordance with 50% represents chance level. Any value that is higher than 50% indicates accordance with the direction of C, whereas any value below 50% indicates accordance with the opposite direction of C., which corresponds to F. Hence, if we subtract 50% from the accordance scale, we can represent data in terms of “Dominance level”. In the dominance-level scale, positive, and negative % values represent the dominance of C and F, respectively. If C and F sum to cancel each other, we expect a 50% accordance level and 0% dominance level.

Results of the experiment are shown in Figure 4b,d. The plots C, F, and CF refer to the three conditions described in Figure 4a. The plot C + F is obtained by adding the results of C and F. As one can see, this simple addition matches the data from the CF condition very well, indicating that the summation is linear.

In summary, these results suggest that non-retinotopic integration does not stem from a high-level decision process by weighing and integrating different response options. Rather, non-retinotopic integration is an unconscious process combining information linearly with accuracy.

### 2.5. The Role of Attention and Mandatory Integration

In the experiments described above, observers were alerted ahead of time as to which stream to attend in order to report the perceived Vernier offset. As shown, integration only occurred within the attended stream. This raises the question as to whether stream-selective integration occurs because of attentional selection or whether integration is independent of attentional allocation. To answer this question, Otto et al. [15] used an auditory cue to manipulate attention. An auditory cue indicated whether to report the perceived Vernier offset of the left or the right stream. As in Figure 4, the stimulus consisted of a central Vernier (C) and a flanking anti-Vernier (F).

**Figure 5:**
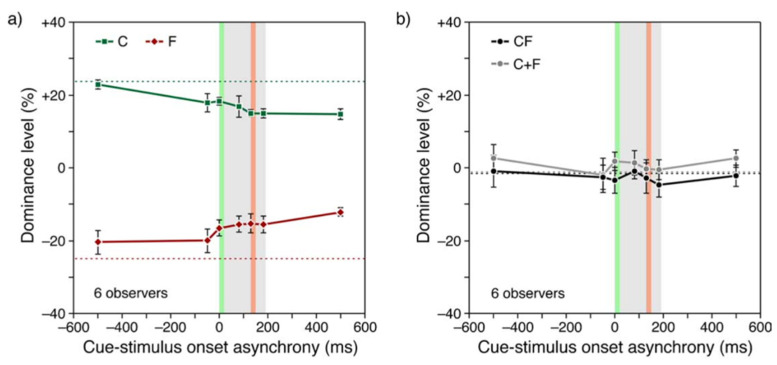
(**a**) Similar paradigm as in the previous figure. In condition C, the central line was offset and there was a line/flank offset in either the left or right stream, randomly chosen in each trial. In condition F, only the line was offset. The *x*-axis shows an auditory cue was displayed indicating to which stream to attend. Negative values indicate pre-stimulus presentation of the cue. Positive values indicate post-stimulus presentation. Hatched, horizontal lines indicate baseline conditions without the cue. Unsurprisingly, dominance decreases the later the cue is presented, i.e., performance decreases. Importantly, had observers been unable to keep the offsets separate in memory, dominance would be at 0. (**b**) When both offsets are in the same stream, they integrate even when the cue appears 500 ms after stimulus onset. Hence, integration occurs pre-attentively. From Ref. [15].

The results are shown in Figure 5, which plots dominance level as a function of Cue-stimulus onset asynchrony (C-SOA). Negative and positive C-SOA values correspond to the conditions where the cue is delivered before and after the stimulus onset, respectively. The gray shaded region in the plots indicates the time interval during which the stimulus is presented with the green and red bands corresponding to the presentation times of C and F, respectively. Delivering the cue during or after stimulus presentation causes a slight reduction in dominance levels (Figure 5a). However, even when the cue is delivered 500 ms after the stimulus onset (i.e., 300 ms after stimulus offset), it does not affect the relative levels of dominance as shown in Figure 5b (CF) nor the linearity of integration (Figure 5b, C + F). Hence, non-retinotopic feature integration does not require unifocal attention. Furthermore, the perceptual outcome does not depend on whether the cue was delivered in temporal proximity of C or F. If integration were selective according to attentional allocation, a cue occurring in temporal vicinity of C or F would lead to the dominance of C or F, respectively. The results do not agree with this expectation and suggest instead that the integration is mandatory. This finding is in stark contrast with “feature binding” hypothesis, i.e., the combination of *different* features, such as color and shape, of an object [16], which require attentional allocation.

Additional evidence for mandatory integration derives from experiments where observers were asked to report *only* the offset of the C or F. Observers were unable to do so. They perceive only one integrated offset, which they were able to report [17]. Hence, decisions are not based on a single Vernier offset, arbitrarily chosen by the observer, but on a mandatorily integrated offset.

### 2.6. “Object Identity” Established by Gestalt Grouping Controls Non-Retinotopic Feature Integration

Integration is spatially precise, grouping matters, and object identity is key. For example, there is no spill-over of Vernier offsets from the attended to the non-attended stream. Small changes in the spatial layout qualitatively change integration (see for example Figure 4 in [8]). Hence, what matters is whether the Vernier offsets are in the same stream, i.e., whether they belong to the same object, as mentioned above.

SM contains a non-retinonotopic component and has a sophisticated memory structure. Evidently, integration is non-retinotopic since integration occurs across space and time. However, there is much more to this process. When elements were removed from the stream so as to disrupt spatiotemporal continuity, there was no integration [18] since there is no object identity. The removal of elements leads to the perception of two different streams, one after the other with separate offsets. In this case, observers were able to report Verniers individually and the integration became impossible. When, however, elements were occluded instead of being removed, integration did occur. Hence, Vernier offset information is held unconsciously in the SM and glued together when object identity is established [18]. This is even more evident in the experiment where observers were asked to make a saccade during the SQM. Hence, due to the saccade, the first part of the stream was at a different location on the retina than the second part. Notwithstanding this eye movement that effectively split the stream to two different locations on the retina, only one stream was perceived, and integration was mandatory, i.e., the brain brought together the information from the two streams and integrated the offsets automatically. This result is maybe less surprising than it may seem at first glance. Imagine that a train is passing with some writing on it. If you make an eye movement, you still see a moving train with a writing on it even though parts of the train are projected to highly non-contiguous retinal locations.

As shown in Figure 2, the contents of *r*SM are susceptible to visual masking while those of *nr*SM are not [3,4]. The post-saccadic stimulus effectively masks the contents of *r*SM, thereby preventing inappropriate integration across different retinotopic locations. On the other hand, as shown above, *nr*SM preserves object identity across saccades and facilitates an accurate and precise integration during ecological vision, a necessity since humans perform 3–4 saccades per second under natural viewing conditions.

### 2.7. Spatio-Temporal Extent of Feature Integration

An inspection of Figure 4 suggests that while dominance levels drop with increased distance between C and F, this drop is relatively mild. The separation between the central Vernier and the flank-offset placed in position 5 is 16.5 arcmin.

Temporally, retention-time for *nr*SM is long, and lasts in the range of almost half second. A central Vernier offset and an opposite offset at one of the subsequent lines were presented. The experiment investigated the point at which, for the line of the stream, integration was still mandatory, i.e., integration occurred. This was the case for up to 450 ms, depending on the observer. Offsets from 490 ms on could be reported individually [17]. Hence, *nr*SM has a processing window, which starts with stimulus onset and terminates at around 400 ms. Offsets are only integrated when they are in the same stream. A central Vernier offset, an opposite offset at 330 ms, and one in the same direction as the central Vernier at 590 ms were presented [17]. Observers were asked to report a first and a second perceived offset. When asked about the first offset, observers reported the integrated offset. When asked about the second, it was identified as a single later offset. Importantly, the first two offsets were separated by an ISI almost twice as long as the second and third offset. Hence, what matters is not temporal proximity between Verniers but the belonginess to a stream, which we also called a window of integration previously [19].

## 3. Can *r*SM Be Dropped and Replaced Completely by *nr*SM?

**Figure 6:**
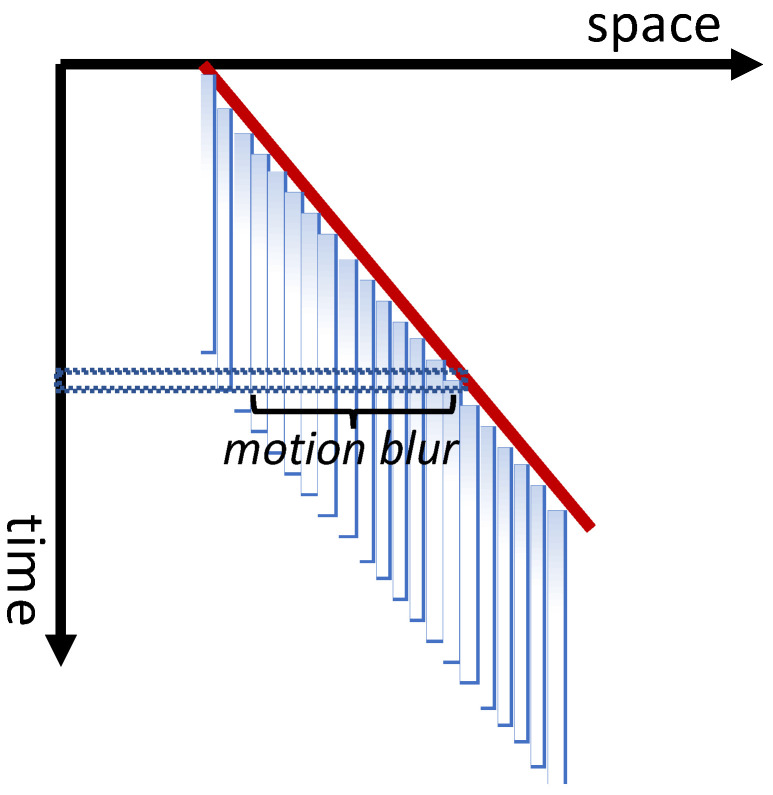
Illustration of motion smear that would result from rSM in response to a moving stimulus. A dot moves at a constant speed (red line) as plotted in the space-time diagram. At each retinotopic location, it generates a decaying activity, as illustrated by fading blue lines. The spatial profile at a given time (dashed horizontal area) consists of the stimulus and a trailing motion blur similar to a comet and its tail. Note that different colors do not refer to the color of the perception but are used to illustrate different components.

Given the evidence reviewed above supporting *nr*SM, one can ask whether *r*SM is still required and whether a single non-retinotopic memory mechanism can account for the extant data. The answer to this question can be found in studies that examined the perception of blur for moving targets. The two sensory memory mechanisms provide different predictions on whether we should perceive blur for moving targets. The reference frame for *r*SM is retinotopic. Hence, a moving stimulus will activate neighboring retinotopic locations successively and briefly. As the stimulus moves to a different retinotopic location, the activity generated at the current retinotopic location will start to decay accordingly. As shown in Figure 6, this, in turn, will generate a gradual decaying activity behind the moving target, such as the “tail” of a comet.

In fact, this prediction can be verified easily in dark-adapted conditions with a small flashlight acting as the stimulus. As we move the flashlight, we perceive the flashlight along with a “tail” and if we move the flashlight in a periodic pattern, such as a circle, fast enough so that the stimulation reaches its starting point before the decay of activity there, a complete figure corresponding to the periodic trajectory of the stimulus (e.g., a circle) can be readily perceived. This is because scotopic vision is driven by rods that have temporally sluggish responses. With regard to photopic vision, Burr [20] presented subjects with a random array of dots that moved for various exposure durations. The observers’ task was to report the length of perceived motion blur by adjusting the length of a static line-segment. The results showed that for short exposure durations, blur commensurate with SM dynamics was perceived; however, for exposure durations of longer than ca. 30 ms, the perceived blur decreased, and the stimulus appeared sharp for exposure durations ca. 100 ms and longer. These results can be interpreted in favor of eliminating the *r*SM altogether since, with enough exposure duration, the stimuli can activate motion-based reference frames and be perceived without blur. However, Burr’s data were somewhat puzzling because several prior studies reported the perception of motion blur [21,22,23,24,25]. In order to reconcile these apparently contradicting findings, Chen et al. [26] used a stimulus and task similar to the ones used in Burr’s study, however, with an additional independent variable, viz., the density of the dots in the display. Their results showed that decreasing the density of the dots led to increased motion blur and, with low density displays, perceived blur increased monotonically with exposure duration to reach levels commensurate with an *r*SM, with a retention time of about 100 ms [26]. Chen et al. explained the effect of dot density by noting that at high-dot densities, neighboring dots fall in spatiotemporal proximity to produce strong metacontrast masking [26]. As depicted in Figure 2, *r*SM is sensitive to visual masking whereas *nr*SM is not; in other works, the contents of *r*SM can be interfered with using a mask (e.g., reduced visibility, recognition) whereas those of *nr*SM cannot [4]. In fact, the central Vernier in the stimulus of Figure 3 is invisible because it is masked by the flanking Verniers. Masking by flanking Verniers render the contents of *r*SM at the central location invisible; yet the Vernier offset information stemming from this masked central element is robust to masking and becomes effectively visible by being integrated to the Vernier of the flanking elements in the motion stream. In summary, we propose that *r*SM and *nr*SM are two parallel and complementary memory sub-systems. The former is tuned to static stimuli whereas the latter to dynamic stimuli. During fixation or smooth pursuit, the stabilized target can be stored in *r*SM after its offset. For dynamic stimuli, the spatiotemporal transients “turn off” *r*SM by masking its contents. On the other hand, *nr*SM remains active and processes stimuli according to motion-based non-retinotopic reference-frames. In the next section, we highlight differences in the temporal dynamics of these two memory sub-systems and place the quantitative findings in the context of their respective “ecological niche”.

## 4. A Quantitative Analysis of Memory Dynamics and Its Computational Implications

### 4.1. Ternus-Pikler Displays and Nonretinotopic Feature Integration

**Figure 7:**
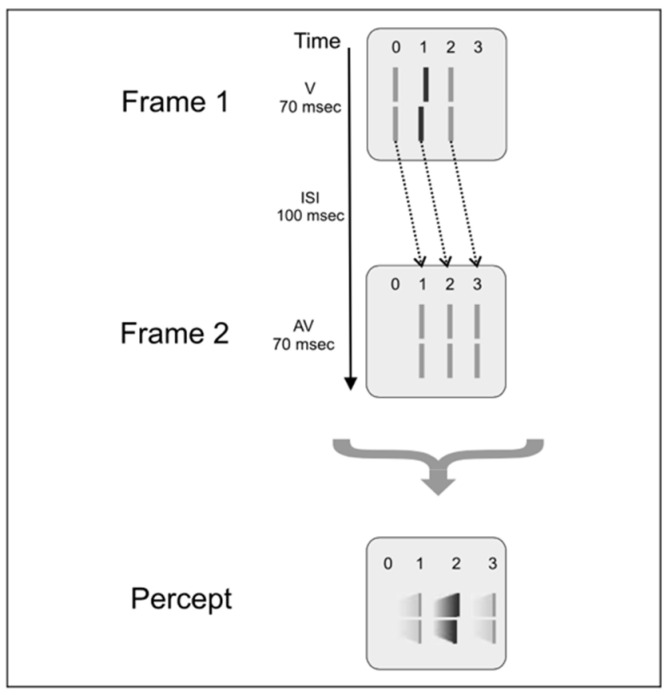
Ternus Pikler Display. Three lines presented, with only the central line being offset. An ISI of 100 ms followed and then another 3 lines were presented, all non-offset and shifted one inter-line spacing to the left. Surprisingly, the first frame offset is not perceived at the left most line in the second frame but at the central line—reflecting that integration respects object identity and occurs not in retinotopic coordinates. Reprinted with permission from ref. [12]. Copyright 2021 MIT Press.

A typical Ternus-Pikler display [27,28] consists of two frames, separated by an Inter-Stimulus Interval (ISI). As shown in Figure 7, typically, the first frame contains three elements, and the second frame consists of the same stimulus shifted by one inter-element distance. In Figure 7, the elements are Vernier stimuli. As in sequential metacontrast, a Vernier offset is introduced to select elements in the display and observers are asked to report the perceived Vernier offset for elements in the second frame (Figure 7). The way observers perceive this stimulus depends on the ISI. In brief ISIs, the common elements in the two frames (elements marked 1 and 2 in Figure 7 appear stationary whereas element 0 appears to move to element 3. This percept is called “*element motion*” [29]. When the ISI is long, all three elements appear to move together with the following motion correspondences: 0→1, 1→2, and 2→3, as depicted by the dashed arrows in Figure 7. This percept is called “*group motion*” [29].

**Figure 8:**
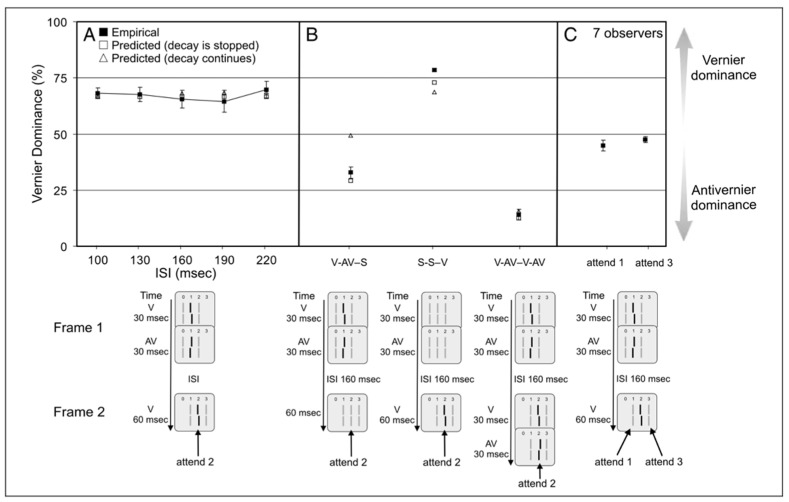
(**A**). A vernier offset was followed by the AV at the central position in frame 1. In Frame 2, the corresponding middle position had a V. We varied the ISI between the frames. Performance is roughly the same, which indicates that, after the disappearance of frame 1, the contents of the memory remain constant and do not decay. (**B**) We replaced some offsets (V and AV) by straight lines (S). In V-AV-S, the AV dominates as expected from leaky integration. This dominance increases further when, as in frame 2, a V-AV pair is also present. (**C**) Control condition showing that integration is stream specific. When observers attend and report the first and third elements of the last frame, there is no retinotopic integration. The open triangles show predictions of the leaky-integrator model. Open squares show the predictions for the same model with the only exception that the decay term (the first term on the right-hand side of Equations (1) and (2)) is removed during the ISI. In other words, in this alternative model, the leak occurs during stimulus presentation but not during ISI. Conditions V-AV-S and S-S-V show better fits with the experimental data for the latter model. Reprinted with permission from ref. [12]. Copyright 2021 MIT Press.

Figure 8 shows a slightly modified version of the Ternus-Pikler stimulus. The first frame is divided into two parts to study retinotopic integration, whereas non-retinotopic integration is studied based on the aforementioned motion correspondences during group motion.

Consider first the leftmost case shown in Figure 8. The stimulus consists of the retinotopic integration of V and AV (the two parts of the first frame) and the non-retinotopic integration of this with V is presented in the second frame. Observers were asked to report the perceived Vernier offset of the central element in the second frame. In the experiment, the ISI varied from 100 ms to 220 ms to investigate the temporal integration time. The results (filled squares in the upper panel) show that integration, as assessed by Vernier dominance, is independent of ISI within this range. The V-AV-S condition, where S denotes “straight” Vernier, i.e., a Vernier stimulus with a zero offset, in panel B, shows that in the absence of V in Frame 2, AV dominates. This demonstrates that, indeed, V in Frame 2 integrates non-retinotopically with the two Verniers presented in Frame 1. When only the V of Frame 2 is presented (S-S-V in panel B), the Vernier dominates as expected. Finally, adding an AV to Frame 2 (V-AV-V-AV in Panel B) leads to strong Anti-vernier dominance. The data in Panel C is used as a control condition to demonstrate that integration is stream specific and as mentioned before, follows the stream specificity set by the Ternus-Pikler group-motion correspondence that binds the central element of Frame 1 to the central element of Frame 2 non-retinotopically (the, 1→2 correspondence).

### 4.2. A Quantitative Analysis of Retinotopic and Non-Retinotopic Feature Integration

To analyze quantitatively the implications of these data, we began with a very simple model of integration and persistence [12]. Both Let $x_{V}\left(t\right)$ and $x_{AV}\left(t\right)$ represent the temporal activities of feature detectors that are tuned to the Vernier and Antivernier offsets, respectively. A first-order constant-coefficient differential equation is one of the the simplest models for temporal integration and persistence:

(1) $$\frac{dx_{V}\left(t\right)}{dt}=-\tau x_{V}\left(t\right)+I_{V}\left(t\right)$$

and

(2) $$\frac{dx_{AV}\left(t\right)}{dt}=-\tau x_{AV}\left(t\right)+I_{AV}\left(t\right)$$

where $I_{V}\left(t\right)$ and $I_{AV}\left(t\right)$ are the Vernier and Antivernier inputs (stimuli), respectively. Since we have the derivative of the feature-detector activities on the left-hand-side of equation, the feature-detector activity consists of an *integration* of these inputs. The term $-\tau x_{V}\left(t\right)$ is a passive-decay term, so that when the input is turned off $I_{V}\left(t\right)=0$ the activity of the feature detector *decays* with time-constant τ to its resting-level, which is zero. This type of equation is widely used in neural modeling and is known as the *additive* or *leaky-integrator* [30] model. The term ‘leaky’ is used to express the decaying nature of the dependent variable in these equations. In the context of memory, the terms “leaky” or ‘leak’ are used to highlight the fact that the informational contents of the memory decay. When V and AV stimuli are inserted into the same retinotopic location or along the same motion-stream, the outputs of these feature detectors are pooled together to generate the overall integrated activity y(t) that combines both V and AV stimuli:

(3) $$\frac{dy\left(t\right)}{dt}=-\tau y\left(t\right)+x_{V}\left(t\right)-x_{AV}\left(t\right)$$

**Figure 9:**
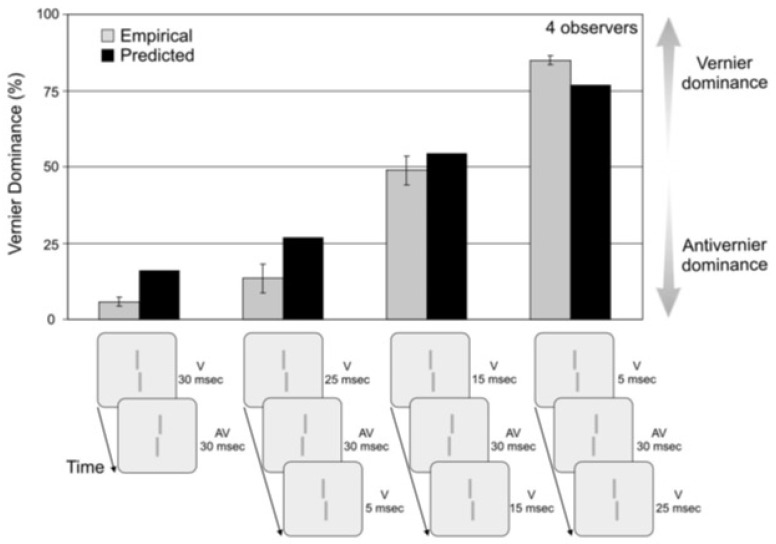
A vernier (V) was followed by a vernier offset in the opposite direction at the location (AV). When the stimulus duration is 30 ms each, the AV strongly dominates. The vernier offset V was presented before and after the AV. We kept the overall duration of the V constant at 30 ms. The longer the after-AV duration, the stronger dominance, i.e., the V contributes more to performance than the AV. Black bars show the predictions of a leaky integrator model. Reprinted with permission from ref. [12]. Copyright 2021 MIT Press.

Since V and AV are opposite to each other and, as the experimental data show, subtract from each other, their difference appears on the right-hand side of the differential Equation (3). To relate this activity to the percept, we used a sigmoid, which is a function well known to characterize psychometric curves, i.e., percent correctness as a function of stimulus strength. The variable y(t) is the internal (neural) representation of stimulus strength and the dominance data admit lower and upper bounds (0% and 100). Hence, the following sigmoid function is used to convert the activity y(t) to the dominance levels shown in Figure 8

(4) $$d\left(y\left(r\right)\right)=\frac{100}{1+\exp\left(-\frac{2y\left(r\right)}{\sigma }\right)}$$

where r is the “read-out time”, i.e., the time at which the contents of the integrated activity are read to report the perceived Vernier offset and the constant σ determines the slope of the sigmoid function. By using three free parameters (r, τ,σ), this model was fitted to the data shown in Figure 9, as well as 30 other combinations of V and AV sequences.

Figure 9 shows the predictions of this model for retinotopic integration. Overall, the model captured well and yielded a decay rate constant τ=0.0291 ms^−1^, corresponding to a time constant $\frac{1}{\tau }=34.36$ ms. This is a relatively fast decay rate commensurate with the temporal dynamics of retinotopic SM. Note that the decay-rate of SM is not fixed, and depends on stimulus parameters, such as background and stimulus luminance [31]. When this model is applied to non-retinotopic integration data (open triangles in Figure 8), it fails to capture the dynamics of non-retinotopic integration. For example, for the V-AV-S condition, the model predicts a dominance level of close to 50%, whereas the data indicate an anti-Vernier dominance of ca. 30%. This is because, due to the relatively fast decay of retinotopic memory, the integrated activity from Frame 1 decays to baseline during the 160 ms ISI. In order to account for the storage of information during the ISI, an alternative version of this model, in which the decay is stopped after the offset of the first frame was also tested (open squares in Figure 8). By “decay is stopped”, we mean that the contents of the memory stay constant and do not deteriorate due to decay. This is achieved by removing the decay, or the leak term, $-\tau x_{V}\left(t\right)$, from the equation during the ISI. Since, during the ISI, the input $I_{V}\left(t\right)=0$, Equation (1) becomes $\frac{dx_{V}\left(t\right)}{dt}=0$, i.e., $x_{V}\left(t\right)=constant$. The same applies to Equation (2). This model was successful in explaining the non-retinotopic storage of information. Hence, *r*SM and *nr*Sm have different time-constants and different temporal dynamics.

### 4.3. Retinotopic and Non-Retinotopic SM

**Figure 10:**
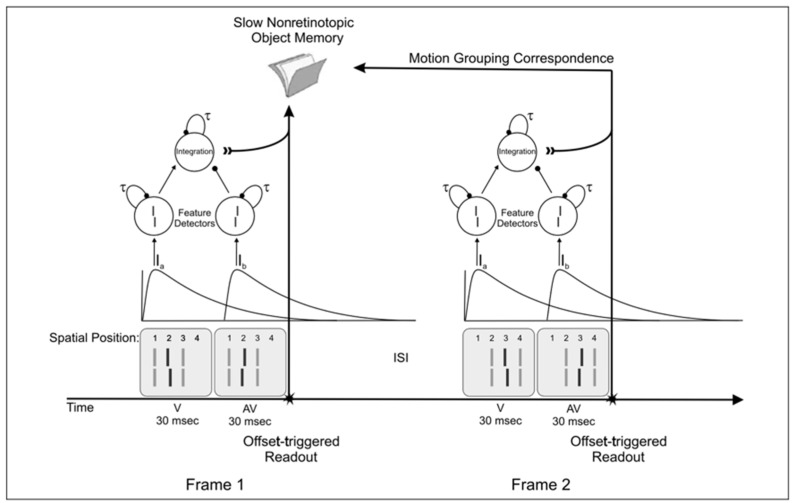
A model for a non-retinotopic object memory and integration. For each frame and retinotopic location, there is an integration neuron, which gives more weight to the second offset. When the first frame disappears, the integration is terminated, and the content stored in memory. It is reopened when the second frame is terminated. Integration occurs according to object correspondence, i.e., non-retinotopically. Reprinted with permission from ref. [12]. Copyright 2021 MIT Press.

Figure 10 schematically illustrates the operation of the modified model described in the previous section.

The *V* and *AV* information in the first frame is integrated retinotopically with a time-constant of 34.36 ms, and this integrated information is “frozen” at the end of Frame 1 (stopped decay, possibly triggered by the offset transient). As discussed in the previous section, this would be equivalent to removing the decay term from the differential equation. Mechanistically, this can be achieved by introducing a modulatory transient-driven signal, m(t), such that m(t) = 0, when a transient signal is triggered and m(t) = 1, otherwise:

(5) $$\frac{dx_{V}\left(t\right)}{dt}=m\left(t\right)\left[-\tau x_{V}\left(t\right)+I_{V}\left(t\right)\right]$$

(6) $$\frac{dx_{AV}\left(t\right)}{dt}=m\left(t\right)\left[-\tau x_{AV}\left(t\right)+I_{AV}\left(t\right)\right]$$

(7) $$\frac{dy\left(t\right)}{dt}=m\left(t\right)[-\tau y\left(t\right)+x_{V}\left(t\right)-x_{AV}\left(t\right)$$

When the second frame is presented, the non-retinotopic reference-frame groups’ elements correspond at position 2 of Frame 1 in Figure 8 with those at position 3 of Frame 2 (see Figure 7 for element correspondences in the Ternus-Pikler display). Then, integration occurs between the elements in the two frames. This in turn can map the modified model of SM as follows. When the first frame is presented *r*SM and *nr*SM receive information about the first V and AV and an integration with time-constant of 34.36 ms takes place. This retinotopic information occurs automatically in *r*SM and can be conveyed from *r*SM to *nr*SM or *nr*SM, with an integration time-constant that depends on the reference-frame. Additional work is needed to distinguish between these alternatives. A major difference between *r*SM and *nr*SM is that the information in *r*SM decays automatically, whereas information in *nrSM* does not because it relies on motion-grouping based reference frames. A motion-grouping based system operates within a time-window tuned to the spatiotemporal dynamics of *motion.* These spatiotemporal properties are expressed by Korte’s third law [32].

**Figure 11:**
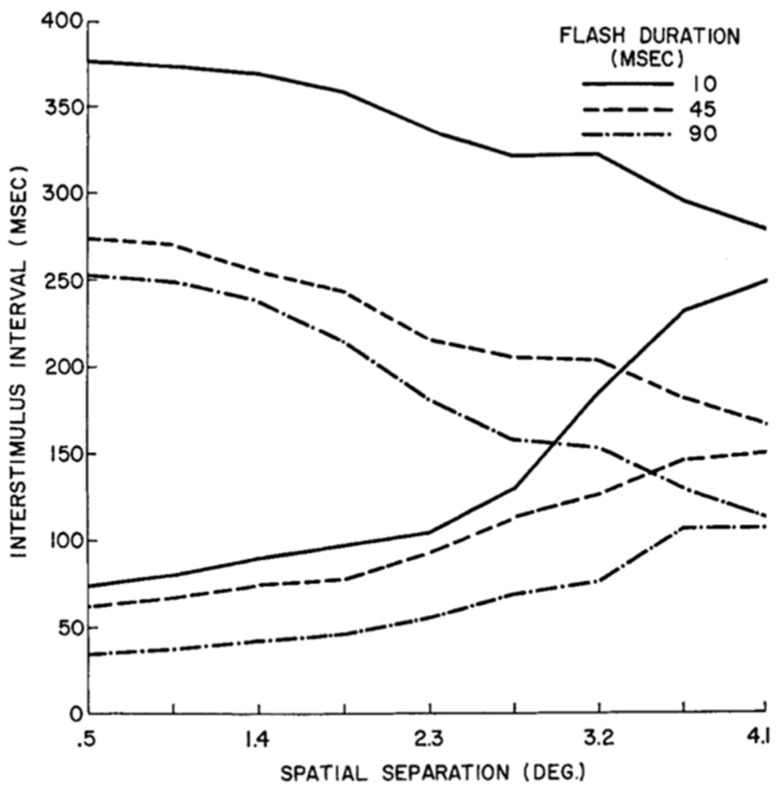
Two spatially separated flashes can be perceived as occurring simultaneously, in apparent motion, or as separate flashes appearing in succession. The figure shows these three distinct percepts as a function of flash spatial (*x*-axis) and temporal (*y*-axis) separation for three different flash durations. For each duration, simultaneity and succession are perceived below the lower curve and above the upper curve, respectively. Between these two curves, apparent motion is perceived. From Ref. [33] with original data from Ref. [34].

Figure 11 plots the way successive flashes are perceived as a function of flash duration, their spatial separation, and the temporal interval between the two flashes. Two curves are plotted for each flash duration. Below the lower curve, the two flashes are perceived as simultaneous whereas above the upper curve they are perceived as two sequential flashes without apparent motion. Between the two curves, observers perceive a smooth motion from the first flash to the second. Since *nr*SM’s reference-frame is motion-based, it operates within a spatiotemporal range that is determined by the curves in Figure 11.

In summary, unlike *r*SM, in which integration occurs within a narrow retinotopic range and a narrow temporal range, *nr*SM can operate over more extended spatial and temporal ranges. We attribute this fundamental difference to the fact that the reference-frame of *nr*SM is motion-based and hence its spatiotemporal properties are in accordance with those of the motion system.

## 5. Discussion and Conclusions

In this paper, we argued that the SM component of the Atkinson–Shiffrin model needs to be updated to account for ecological viewing conditions. Since SM is the first stage of memory, from which the rest of the components receive inputs, this puzzle questioned the validity of the entire Atkinson–Shiffrin model questionable. To address this puzzle, we previously proposed a modification of SM by integrating a non-retinotopic component. In this paper, we carried out an analysis of extant data in order to highlight differences between *r*SM and *nr*SM. The traditional view of SM is as a passive, rapidly decaying store that can be characterized mathematically by an exponential decay triggered at stimulus offset. We showed that this model fails to explain the dynamics of *nr*SM. The operation of *nr*SM is complex and involves processes such as motion-based Gestalt grouping and motion-based reference-frames. Hence, we suggest that the spatiotemporal dynamics of *nr*SM are in accordance with the spatiotemporal dynamics of the motion system.

One important finding is that eye movements do not interrupt ongoing integration in the *nr*SM. Integration in the SQM occurs even when observers make a saccade. What matters is object identity in the external world. It is clear that integration in the SQM cannot be explained by a passive, non-retinotopic, low-level mechanism with time constants shorter than 150 ms. In Table 1, we summarize the major differences between *r*SM and *nr*SM.

In addition, classic models of decision making need to be rethought, since decisions were made only after accomplishing integration in the *nr*SM. First, the *nr*SM represents a long-lasting buffer, in which information is integrated, before it is submitted to a drift-diffusion process (Figure 7, [35,36]). Second, information obtained later weighs stronger than that presented earlier (Figure 7), which is not easy to reconcile with classic models, in which earlier stimuli drive the evidence. Similar arguments apply to the classic, feedforward model of vision, including deep neural networks (DNNs), which seem to miss dynamic components of integration, as they occur in the *nr*SM.

## Figures and Tables

**Table 1:** Comparison of retinotopic and non-retinotopic sensory memory.

Sensory Memory Type	Reference-Frame	Retention Period	Maskable?	Sensitive to Gestalt Grouping and Object Identity?	Robust Across Saccades?	Tuned For
***r*SM (traditional sensory/iconic memory)**	Retinotopic	Varies with stimulus conditions such as background luminance but in general in the order of 100 ms	Yes	No	No	Static (or stabilized) stimuli during fixations and smooth pursuit
***nr*SM**	Non-retinotopic	Longer than *r*SM and can extend to ca. 450 ms	No	Yes	Yes	Dynamic stimuli, in particular moving stimuli that preserve their spatiotemporal identity
